# An Inclusive Leadership Framework to Foster Employee Creativity in the Healthcare Sector: The Role of Psychological Safety and Polychronicity

**DOI:** 10.3390/ijerph19084519

**Published:** 2022-04-08

**Authors:** Qinghua Fu, Jacob Cherian, Naveed Ahmad, Miklas Scholz, Sarminah Samad, Ubaldo Comite

**Affiliations:** 1 Department of Business Administration, Moutai Institute, Renhuai 564507, China; 2016101050084@whu.edu.cn; 2 College of Business, Abu Dhabi University, Abu Dhabi P.O. Box 59911, United Arab Emirates; jacob.cherian@adu.ac.ae; 3 Faculty of Management Studies, University of Central Punjab, Lahore 54000, Pakistan; 4 Faculty of Management, Virtual University of Pakistan, Lahore 54000, Pakistan; 5 Division of Water Resources Engineering, Department of Building and Environmental Technology, Faculty of Engineering, Lund University, P.O. Box 118, 221 00 Lund, Sweden; 6 Department of Civil Engineering Science, School of Civil Engineering and the Built Environment, University of Johannesburg, Kingsway Campus, P.O. Box 524, Aukland Park, Johannesburg 2006, South Africa; 7 Department of Town Planning, Engineering Networks and Systems, South Ural State University (National Research University), 76 Lenin Prospekt, 454080 Chelyabinsk, Russia; 8 Institute of Environmental Engineering, Wroclaw University of Environmental and Life Sciences, ul. Norwida 25, 50-375 Wrocław, Poland; 9 Department of Business Administration, College of Business and Administration, Princess Nourah Bint Abdulrahman University, Riyadh 11671, Saudi Arabia; sarminasamad@gmail.com; 10 Department of Business Sciences, University Giustino Fortunato, 82100 Benevento, Italy; u.comite@unifortunato.eu

**Keywords:** leadership, creativity, psychological safety, healthcare system, polychronicity

## Abstract

Creativity at the level of employees is of utmost importance for every sector of an economy, with no exception to a healthcare system. The reason why employee creativity is important lies in the fact that employees have profound knowledge of their job and thus can serve as a source of meaningful innovation in an organization. Research shows that employee creativity is largely dependent on leadership. Corporate leaders significantly influence subordinates’ behavior. However, with the economic development, globalization, and changing business environment, a traditional authoritative leadership style can no longer be effective in understanding employees’ psychological needs to foster their creative behavior. In this regard, the role of inclusive leadership as an effective organizational management strategy was recently discussed in literature at different levels. It was also stated that an inclusive leader could foster employee creativity. However, such relationships in healthcare systems of developing economies have largely remained under-explored previously. We explored employee creativity in a healthcare context of a developing economy in an inclusive leadership framework to bridge such knowledge gaps. We also investigated the mediating roles of psychological safety and polychronicity in the above-stated relationship. We collected the data from hospital employees through a questionnaire (paper–pencil method). A hypothetical model was developed, which was tested through structural equation modeling in AMOS. Based upon the statistical outcomes, we found that an inclusive leadership style in a hospital can significantly foster employee creativity, whereas psychological safety and polychronicity mediate this relationship. This study offers different theoretical and practical insights, especially to a healthcare system. An important finding was that an inclusive leader can motivate the followers to be more creative. This finding is significant for a hospital because creative employees provide a hospital with a solid competitive base.

## 1. Introduction

In today’s corporate environment, which is very competitive and challenging, creativity plays an important role in the success of an organization [1]. Creativity in the context of a workplace keeps an organization moving forward successfully. Innovation as an outcome of employee creativity is of high value for an organization as such innovation is meaningful because employees have a better knowledge of their workplace and job of how to perform their work in creative ways. Despite the fact that massive development in the global healthcare system has been evident during recent years, both national and global health systems are identified as “resource deficient” [2]. In this respect, healthcare systems around the globe are continuously trying to optimize their resources through process-driven advancements, standardized procedures, and innovations in order to reduce costs while improving the quality of services. Whether operating in public or private spheres, hospitals prioritize serving their patients in creative ways. To this end, from an efficiency and effectiveness perspective, employees of a healthcare organization can serve as a “valued source of creativity” [3] who can lead their organization towards success.

Similar to most businesses, hospitals also face a dynamic market environment characterized by constant change and erratic challenges [4]. To elucidate further, the healthcare sector faces a constant pressure situation that demands creative service delivery solutions to outperform the rivals on one hand and get an attractive place in the minds of clients (the patients) on the other hand. The above discussion clearly indicates the significance of seeking innovation, incrementally or radically, as an outcome of creativity on the part of employees. A review of related literature uncovers that most of the prior literature investigated the outcomes of employee creativity. For example, it was realized that employee creativity could boost the performance of an organization [5,6] or place an organization in a better competitive position [7]. From an economic perspective, the above studies were important. Nevertheless, we take a different position to advance the debate on employee creativity. That is, to answer what drives employee creativity in an organization? Though the early work identified some factors of employee creativity, for example, job autonomy [8], psychological capital [9], perceived organizational support [10], organizational culture [11], and leadership [12] may influence employee creativity. However, the inconsistent results indicate that a consensus has not yet been reached. Furthermore, employee creativity in healthcare did not receive due attention previously. Hence, there is a dire need to find out the factors that drive employee creativity in the healthcare segment. Therefore, one of the critical aims to carry out this study is to find out the factors that influence employee creativity.

Research shows that different factors influence individual behavior. It was mentioned that various organizational factors affect employee behavior in an organizational context. In this regard, it was realized that an effective leadership style, as an organizational factor, can drive employees’ behavior in a workplace [13]. The role of inclusive leadership to foster employee creativity was recently discussed at different levels [14,15]. The greater focus of an inclusive leader on openness is something that places this leadership style at the heart of employee creativity [16]. Although the role of inclusive leadership is well discussed in literature from a perspective of employee creativity, surprisingly, the healthcare sector remained an understudied area. Given that employee creativity is a matter of prime concern to this sector, investigating the role of inclusive leadership to enhance employee creativity is worthwhile. Therefore, this study also aims to investigate the relationship between inclusive leadership and employee creativity in a healthcare context. In this regard, we propose the following hypothesis.

**Hyphothesis** **1.**  *An inclusive leader gives rise to employee creativity in an organization.*

Another factor driving employee creativity in an organizational context is psychological safety (P.S) [17,18]. Although the concept of P.S existed in organizational science for a long time, empirical research in this domain has been flourishing recently [19]. Indeed, P.S is an employee’s belief that he/she will not be punished or humiliated by others in a workplace for sharing different ideas, questions, or concerns [20]. Extending this discussion, we refer to a two-year report published by Google on discovering the most influential factors for a great team. The findings of this document were interesting as the most significant factor of a great team was not the individuals with the highest IQs or people with vast experience in a field. Rather, it was found that it is employees’ perceptions that they work in a psychologically safe organizational environment. Another interesting finding of the survey was the team that made mistakes was more successful. The underlying reason for this result was to create an environment in which employees feel psychologically safe to take risks, which is critical to fostering creativity and innovation. It has been found that the support from a leader in an organizational context can stimulate subordinates’ motivation to get engaged to show their creative potential [21]. An effective leader develops a work environment characterized by a high-quality leader–member exchange of relationships [22], empowers employees to think differently and builds trust [23]. This latter point (trust) is well discussed in the literature to shape employees’ behavior, especially their creative behavior [24,25,26], highlighting that P.S has a clear role here. As an outcome of leadership support, this perception of working in a safe environment puts employees at ease to engage in creativity without fear [27]. Thus, an effective leadership style promotes P.S in an organization [28]. Though the literature argues about the positive link between P.S and employee creativity, its mediating role in the inclusive leadership framework was not realized, especially in healthcare. Hence, investigating the mediating role of P.S between inclusive leadership and employee creativity is another objective of this study. The above discussion may be summarized by proposing the following hypotheses.

**Hyphothesis** **2.**  *Inclusive leadership style can have a direct impact on psychological safety perceptions of employees.*

**Hyphothesis** **3.**  *Psychological safety mediates between inclusive leadership and employee creativity.*

Literature also highlights different personal factors to spur employee creativity in an organizational context [29,30]. In this vein, a personal factor that recently entered into the lexicon of individual creativity is polychronicity, an individual’s preference for multi-tasking [31]. However, the mediating role of polychronicity for employees’ behavior formation, especially their creative behavior, was not realized. We feel that polychronicity is related to one’s preference for multi-tasking, which can further support an employee to be engaged in creative tasks. Literature shows polychronicity is largely influenced by culture [32,33] and the milieu in which employees interact with their workplace environment. It was also mentioned that the polychronicity of employees is influenced by different organizational factors, including leadership [34,35]. In a healthcare context, few studies investigated the direct impact of polychronicity on employee creativity [36]. Nevertheless, its mediating effect on the relationship of inclusive behavior and employee creativity was not discussed. Thus, the last objective of this study is to test the mediating role of polychronicity between inclusive leadership and employee creativity. In this respect, we propose the following hypotheses.

**Hyphothesis** **4.**  *Employees’ polychronicity can be linked positively with their creativity.*

**Hyphothesis** **5.**  *Employees’ polychronicity mediates between inclusive leadership and employee creativity.*

The target segment of this study was the healthcare sector of Pakistan, which is a developing nation. To represent the healthcare sector, we select hospital organizations that operate in the country with a mix of private and public. Considering the competitiveness in this sector [37] and changing preferences of patients, a hospital must differentiate itself meaningfully from. From this perspective, employee creativity could be a way forward for a hospital. Furthermore, every hospital’s standard operating procedures are the same and are governed by a regulator [38]. Because these procedures to handle a patient are standardized, it implies that not much room is available for a hospital to differentiate itself from the rest of the crowd. Furthermore, the physical outlay of a hospital and the way services are delivered can also be imitated easily by a rival. Thus, finding a meaningful differentiation in this sector is a matter of concern for a hospital. The above situation clearly highlights the importance of employee creativity in this sector, as innovation as an outcome of employee creativity is hard to imitate because it is idiosyncratic in detail. Thus, creative employees can place a hospital in a better competitive position. To boost employee creativity in this sector, the role of leadership is important to create a workplace environment in which employees are motivated to show their creative potential. However, the role of inclusive leadership with this aspect, as an effective management strategy, was not investigated earlier in this sector.

**Figure 1:**
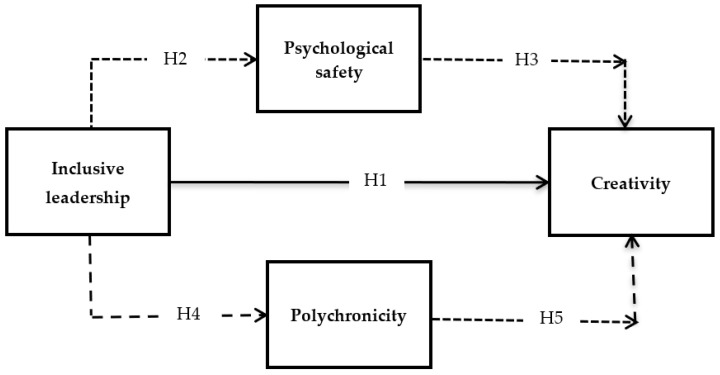
Hypothetical Framework.

The theoretical roots of this study are based on social exchange theory (SET) which was developed by Homans [39]. The early work of Choi et al. [40] and Qi et al. [15] also employed this theory to explain how the process of social exchange between an inclusive leader and a follower urges him to engage in different extra-role behaviors, including creative behavior. An inclusive leader not only encourages openness in a workplace, he/she also promotes a workplace culture of fairness, trust, respect, and collaborations with the followers. At the same time, such leaders also help their employees in situations that do not come under a formal contract of employees. When employees receive supporting and caring conduct from their inclusive leader, they are urged to provide extra support to their leader in the process of social exchange. Consequently, they engage in different extra-role behaviors, one of which is their creative potential. The theoretical framework of this study is given in Figure 1.

## 2. Methodology

### 2.1. Participants and Procedure

The healthcare sector of Pakistan was considered for the hypothetical framework of this study. The country’s healthcare system is a mix of different players, including public, private, charity-based contributors, parastatal, etc. There are four major modes of healthcare delivery in Pakistan: preventive, curative, promotive, and rehabilitative healthcare services. Approximately eighty percent of the country’s population is attended by the private sector [41]. Currently, the provincial government of each province is constitutionally responsible for regulating the healthcare system and structure in a province except the territory administered by the Federal government. The Ministry of National Regulation and Services is a body that sets the policy guidelines of the healthcare system in the country. Nevertheless, the operationalization of the guidelines provided by the Ministry of National Regulation and Services lies with the provincial governments. Large cities of Pakistan, especially Lahore and Karachi, are identified as the two dominant cities where many hospitals exist (both public and private). Moreover, these two cities comprise a multi-million population whose health delivery is reliant on these hospitals. Furthermore, with the rising competitive norms in the healthcare industry, especially in private hospitals, a hospital needs to base its competitive position on a stable foundation, for which creative employees are critical.

Given that Lahore and Karachi constitute a large umbrella of hospitals, we selected these two cities for the purpose of data collection. In this respect, different hospitals were contacted to facilitate the data collection process in the larger interest of industry and academia. We then approached hospitals with a positive response to start the data collection activity. A total of six hospitals were included in the finalized sample (three from each city).

We, prior to producing the final version of the data collection instrument (a self-administered questionnaire), requested the field experts to assess the statements of our questionnaire for their suitability and appropriateness. The significance of this step is endorsed by various researchers previously [42,43]. This expert opinion led us to produce the finalized version of our instrument, which was then presented to each informant [44,45,46]. The employees serving in these hospitals were invited to partake in the current survey on a voluntary basis. Indeed, employees from different departments and fields were included in the current survey. To observe the ethical guidelines, we followed the Helsinki Declaration’s protocols [47,48]. In this regard, the anonymity of each informant was assured, and each informant was served with informed consent to partake in this survey. Furthermore, the quitting from this survey was also allowed if an informant was uncomfortable disclosing the information at any stage in filling the responses on the questionnaire.

### 2.2. Instrument

An adapted questionnaire was considered to collect the data from informants on a seven-point Likert scale. We employed a paper–pencil survey methodology to receive the responses. Generally, the questionnaire included two major sections. The demographic information was collected in the first section, whereas the variable-related information was the subject of the second part. A three-wave data collection procedure was applied in this vein. A time interval of three weeks was maintained for each wave. To elucidate further, in the first wave, the demographic information of the informants was obtained. The information for P.S and employees’ perceptions of inclusive leadership (InL) was also obtained in this phase. Employees with managerial ranks or leadership positions were approached in the second wave to share their perception about a subordinate’s creative behavior. Lastly, the data for polychronicity were collected in the third wave. The early researchers in the field also found this multi-wave data collection strategy as an effective strategy to deal with informants’ fatigue and to avoid the issue of common method variance (CMV), which is a largely reported issue in a survey in which all information was collected from a single source [49].

### 2.3. Measures

To measure the variables of this study, we adapted the already existing scales from different published sources. For example, nine items to measure InL were adapted from Carmeli et al. [50]. A sampled item from this scale was “Our leader/manager is open to discuss the desired goals and new ways to achieve them”. A reliability value (α) of 0.922 was obtained for this scale. In the same vein, we adapted five items of P.S from the study of Edmondson [51]. A significant α = 0.855 was observed in this case. A sample item was “No one in this hospital would deliberately act in a way that undermines my efforts”. The scale of employee creativity was adapted from the stud of Coelho and Augusto [52], which included five items. One item of this scale was “This person experiments with new approaches in performing his/her job”. The overall α = 0.868 showed a significant value.

Lastly, the scale of polychronicity was adapted from Lindquist and Kaufman-Scarborough [53], which included five items with α = 0.869. One particular item was “I prefer to do two or more activities at the same time”. For more details on the items of this survey, Appendix A can be seen. Initially, we distributed 600 surveys to the employees of the selected hospitals who responded with 61% (*n* = 366). For more descriptive detail, Table 1 can be seen. The data were collected between September to November 2021.

### 2.4. Non-Response Bias and Common Latent Factor Test

To assess, if the issue of nonresponse bias exists, we compared the informants who provided full information with the informants who did not provide the full information. It was realized that no significant observable discrepancy has existed, implying that a non-response bias was not a matter of concern. Similarly, though the data were collected from multiple sources in different intervals, we still performed a common latent factor (CLF) test to verify the non-existence of CMV. For this purpose, we drew a measured model in AMOS, which was then compared with another alternate measured model (this model includes a CLF). It was observed that neither a CLF model explained a sheer amount of total variance (more than 50%), nor any significant difference between the standardized factor loadings (>0.2) between the two models existed. These results were enough to confirm that a CMV was not a critical issue in this work which requires any measures to address this issue.

## 3. Results

### 3.1. Establishing Validity and Reliability

To establish the validity and reliability of the variables in this work, we first of all checked the standardized factor loadings (λ) of each item (InL = 9, employee creativity = 5, P.S = 5, polychronicity = 5). Usually, a λ-value > 0.5 is considered good; however, values beyond 0.7 are desirable. Table 2 shows the results of factor loadings along with other values. It can be seen that all λ-values were positive and significant. This implies that all the items showed a good λ-value. We then used these λ-values to calculate each variable’s average-variance-extracted (A.V.E) value. Generally, an A.V.E value > 0.5 for a variable indicates a good convergent validity. It was realized that the A.V.Es for all variables were positive and beyond the standard value of 0.5 (A.V.E for InL = 0.597, E.C = 0.559, P.S = 0.607, and PoL = 0.592). These results clearly indicate that the convergent validity was established in every case, and all the items of one variable were converging on it. Thus, the case of convergent validity was well supported by the statistical findings of the current dataset. Moving forward in the process of construct evaluation, we also assessed the candidature of each variable to prove its composite reliability (C.R). To this aspect, we again considered λ-values to calculate C.R value of each variable. A C.R value not less than 0.7 is normally considered a significant value. In the current case (Table 2), all C.R values were above 0.7, which implies that these values were significant (C.R for InL = 0.930, E.C = 0.864, P.S = 0.865, and PoL = 0.878).

### 3.2. Correlations and Divergent Validity

The validation of variables through A.V.E and C.R values led us to move forward in the process of data analysis. Therefore, we performed a correlation analysis in order to see the value and direction of correlation between different pairs of variables. Table 3 shows the results of correlations. According to these results, a positive and significant correlation was observed between different pairs. To explain further, it can be seen that the pair of InL and employee creativity ꟷE.C showed a positive and significant correlation value (*r* = 0.489, *p <* 0.01). This positive association indicates that these variables co-vary in a positive direction with each other. A similar case can be seen in all other pairs (Table 3). All this implies that correlations were all significant in every case. Likewise, we also tested the divergent validity of all of our studied variables. In doing so, we first calculated the square root of A.V.E (sqA.V.E) of each variable which was then compared with the correlational values. A positive case of divergent validity occurs when the sqA.V.E value of a variable is superior to the correlational values in comparison. Put simply, one could see that the sqA.V.E of InL was 0.773 which was superior to the correlational values (InL ⇔ E.C = 0.489; InL ⇔ P.S = 0.416; and InL ⇔ PoL = 0.278). Similarly, a divergent validity was confirmed for all other variables. Lastly, different measurement models were developed in AMOS compared with the hypothesized model (4-factor). It was revealed that the hypothesized model was the most significant compared to the alternate models. These results are presented in Table 4.

### 3.3. Hypotheses Validation

In the last phase of the data analysis, we tested the hypothetical relationships by employing the structural equation modeling technique (SEM) for which we used AMOS software. As an advanced level technique to analyze the complex models, the data scientists have largely considered this technique, especially to analyze complex models (a model which involves multiple mediations, moderations or both). As a second-generation data analysis tool, SEM provides data analysis with a flexible environment with several advanced features which were not available in traditional regression analysis. To proceed with SEM, we drew a structural model twice. Firstly, the structural model was drawn to observe the direct effects without any inclusion of mediator(s) in this model. This was carried out to see the results of H1, H2, and H4. Table 5 shows the output of this structural model (direct effect model). These results explicitly state that H1, H2, and H4 were statistically valid. For example, the purpose of H1 was to establish a positive link between inclusive leadership and employee creativity. In this regard, we evaluated the results of Table 5 and Table 3. The result of Table 3 showed a positive correlation between an inclusive leader and employee creativity (InL ⇔ E.C = 0.489). Likewise, the regression weight in Table 5 indicates a positive change in employee creativity due to a change in inclusive leadership (beta valueꟷβ1 = 0.476; CR = 15.305; *p* < 0.01). These results provided the needed statistical evidence to accept H1 of this work. Thus, it can be stated in the light of the statistical results that in the presence of an inclusive leader, employees are motivated to be engaged in creativity. The same kind of interpretation can be repeated to arrive at the conclusion that H1, H2, and H4 were statistically significant and hence were accepted.

Secondly, we re-drew the structural model; nevertheless, this time P.S and PoL were included in the model as mediators. To see the significance of mediation, we enabled the bootstrapping option in AMOS. In this vein, we selected a larger bootstrapping sample (2000) to see the mediation effect on employee creativity. At the same time, a biased corrected 95% confidence interval (CI) was also employed in this process. The results of mediation analysis have been reported in Table 6. It was noted that both psychological safety and polychronicity mediated between inclusive leadership and employee creativity (H3: E.C ← P.S ← InL: *β*3 = 0.211, Z-value = 10.655, *p* < 0.01; H5: E.C ← PoL ← InL: *β*5 = 0.173, Z-value = 07.208, *p* < 0.01). Hence, as per the statistical findings, we confirm a mediation role of psychological safety and polychronicity between inclusive leadership and employee creativity. Thus, H3 and H5 were also accepted. Furthermore, the mediation effect explained almost 39% of the change in employee creativity.

## 4. Discussion and Implications

Our research contributes to existing knowledge by filling the following knowledge gaps. First, this study is among the few which approach creativity from an individual perspective. To this end, most of the early work in innovation and creativity was conducted at an organizational level. This line of reasoning is also endorsed in the work of Slåttenetal. [54]. Considering the seminal role of employees in the success of an organization, it was important to highlight their creative role well to position a hospital in the face of competition. A second implication is the consideration of this study for P.S and polychronicity as mediators in a unified model. Although prior literature has discussed the mediating role of P.S in a leadership framework, nevertheless, as per our knowledge, the mediating effects of P.S and polychronicity were not highlighted. Third, this study intends to enrich the field of leadership and organizational management from a healthcare context of Pakistan, a developing economy. To this end, a large body of previous knowledge focused on developed countries or non-healthcare contexts. Considering the culture and context specificity of leadership, it was important to carry out more work in a developing context, rather than trying to generalize the context or culture of developed countries in developing countries.

Furthermore, this research helps the healthcare sector of Pakistan in different ways. For instance, this research study tends to place a hospital in a solid competitive position by engaging its employees in creativity as an outcome of an inclusive leadership style. This implication has special relevance to a healthcare system of a country. Considering the isomorphism in the physical outlay of hospitals and standard operating procedures, it is very challenging for a hospital to find a solid base of competitive advantage because isomorphism in the above factors leads hospitals toward competitive convergence (a situation where all players have access to the same resources). In this situation, employee creativity could be a way forward for this sector, at least for two specific reasons. For example, innovations derived from employee creativity are hard to imitate because such innovation is idiosyncratic in detail.

Another important implication of our study to the field is that it highlights the mediation mechanism of P.S and polychronicity between the relationship of inclusive leadership and employee creativity. Given that, for employees to be creative, they need to build a perception of a safe environment to work. This perception to be safe in a workplace is very important from a creativity aspect because when employees see their workplace as safe, they work without the fear of failure. Working without the fear of failure is central to employee creativity. However, to develop this safety perception among employees, the role of leadership is very important. A leader, especially an inclusive leader, plays a seminal role in creating a workplace environment that is perceived as psychologically safe by employees. As stated earlier, an inclusive leader builds trust, collaboration, and fairness with subordinates. All these factors give rise to their perceptions of working in a safe environment, which eventually influences their creative behavior. In the same manner, polychronicity is very important from the perspective of employee creativity. Importantly, in a healthcare context, where the working environment is dynamic, there is a role of employee polychronicity to be creative. An inclusive leader has a clear role in fostering employee polychronicity, which then guides employee creativity. Therefore, to deal with a competitive environment, a hospital needs to understand the seminal role of leadership style for organizational management.

### Way Forward for Future Studies

This study faces some limitations; however, we feel that these limitations also serve as the base for future studies. First, this study was carried out in two large Pakistan cities, which makes this work’s generalizability a bit weaker. In this respect, it is suggested to consider more cities from other regions of Pakistan. Second, due to different policy reasons, hospitals did not share any list of employees with us. Therefore, it was hard to apply a probability sampling technique that is considered superior to a non-probability sampling technique (a case with this study). Therefore, in future studies, it is suggested to adopt a probability sampling technique, if possible. Third, the nature of the data was cross-sectional, which limits the causality of relationships. Although the proposed relationships were significant, we still suggest that future research studies consider a longitudinal data design.

## 5. Conclusions

The significance of employee creativity has become an important business imperative for all sectors in the current era. Employees capable of developing new ideas are the demand of every contemporary organization, with no exception of a healthcare system. To deal with a changing business environment in the face of competition, promoting employee creativity at all levels in an organization is important. Especially in a healthcare context, which is already identified as a sector with insufficient resources, it is important for a hospital to base its competitiveness on employee creativity, which is meaningful and effective. In this vein, hospital management can benefit from the potential role of an inclusive leadership strategy. An inclusive leader, on the one hand, manages organizational resources effectively, and on the other hand, he/she also promotes employee creativity. Not only is the role of an inclusive leader important for employee creativity, but his/her role is also important to foster P.S and polychronicity in a workplace. To sum up, it is suggested in the light of the above scholarly debate that for effective organization management and to promote creativity at the level of employees, hospital management needs to pay a special focus on inclusive leadership style. To achieve this, we suggest that hospitals develop different training programs on a managerial level with a central focus on inclusiveness.

## Figures and Tables

**Table 1:** Demographic detail of sample.

Demographic	Frequency	%
**Gender**		
Male	223	60.93
Female	143	39.07
**Age group (Year)**		
18–22	52	14.21
23–27	59	16.12
28–32	79	21.58
33–37	71	19.40
38–42	49	13.39
Above	56	15.30
**Experience (Years)**		
1–3	69	18.85
4–6	131	35.79
7–9	107	29.23
Above	59	16.12
**Education**		
12 years	57	15.57
14 years	194	53.01
Masters	115	31.42
**Total**	366	100

**Table 2:** Construct evaluation.

	Λ	λ^2^	S.E	T. Values	E-Variance	AVE	C.R
InL						0.597	0.930
InL-1	0.699	0.489	0.049	14.27	0.511		
InL-2	0.711	0.506	0.047	15.13	0.494		
InL-3	0.720	0.518	0.044	16.36	0.482		
InL-4	0.762	0.581	0.038	20.05	0.419		
InL-5	0.818	0.669	0.036	22.72	0.331		
InL-6	0.822	0.676	0.033	24.91	0.324		
InL-7	0.746	0.557	0.051	14.63	0.443		
InL-8	0.738	0.545	0.039	18.92	0.455		
InL-9	0.913	0.834	0.033	27.67	0.166		
E.C						0.559	0.864
E.C-1	0.718	0.516	0.052	13.81	0.484		
E.C-2	0.829	0.687	0.047	17.64	0.313		
E.C-3	0.758	0.575	0.042	18.05	0.425		
E.C-4	0.716	0.513	0.040	17.90	0.487		
E.C-5	0.712	0.507	0.038	18.74	0.493		
P.S						0.607	0.865
P.S-1	0.868	0.753	0.062	14.00	0.247		
P.S-2	0.719	0.517	0.058	12.40	0.483		
P.S-3	0.706	0.498	0.049	14.41	0.502		
P.S-4	0.730	0.533	0.036	20.28	0.467		
P.S-5	0.716	0.513	0.038	18.84	0.487		
PoL						0.592	0.878
PoL-1	0.717	0.514	0.055	13.04	0.486		
PoL-2	0.744	0.554	0.048	15.50	0.446		
PoL-3	0.829	0.687	0.034	24.38	0.313		
PoL-4	0.813	0.661	0.039	20.85	0.339		
PoL-5	0.736	0.542	0.046	16.00	0.460		

Notes: λ = Item loadings, C.R = composite reliability, ∑λ^2^ = sum of square of item loadings, E-Variance = error variance, InL = inclusive leadership, E.C = employee creativity, P.S = psychological safety, and PoL = polychronicity.

**Table 3:** Correlations and discriminant validity.

Construct	InL	E.C	P.S	PoL	Mean	SD
InL	**0.773**	0.489 **	0.416 **	0.278 **	5.02	0.54
E.C		**0.748**	0.338 **	0.396 **	4.77	0.72
P.S			**0.779**	0.319 **	4.39	0.76
PoL				0.769 **	4.98	0.59

Notes: SD = standard deviation, ** = significant values of correlation, and bold diagonal = discriminant validity values.

**Table 4:** Model fit comparison, alternate vs. hypothesized models.

Model	*χ*^2^/*df*	Δ*χ*^2^/*df*	NFI	CFI	RMSEA
4-factor	1.982	_	0.942	0.949	0.043
3-factor	3.408	1.426	0.876	0.882	0.050
2-factor	3.592	0.184	0.839	0.863	0.057
1-factor	5.082	1.490	0.598	0.604	0.083

**Table 5:** Direct effect structural model results.

Hypotheses	Relationship Nature	Beta-Value (SE)	CR	*p*-Value	CI	Decision
H1: E.C ← InL	+	(*β*1) 0.476 ** (0.0311)	15.305	***	0.563–0.611	Accepted
H2: P.S ← InL	+	(*β*2) 0.422 ** (0.0407)	10.368	***	0.529–0.597	Accepted
H4: PoL ← InL	+	(*β*4) 0.336 ** (0.0492)	06.829	***	0.732–0.744	Accepted

Notes: CI = 95% confidence interval with lower and upper limits, **, *** = significant values.

**Table 6:** Mediation and conditional effects.

Path	Estimates	S.E	Z-Score	*p*-Value	CI	Decision
H3:E.C ← P.S ← InL	(*β*3) 0.211 **	0.0198	10.655	***	0.363–0.404	Accepted
H5: E.C ← PoL ← InL	(*β*5) 0.173 **	0.024	07.208	***	0.299–0.369	Accepted

Notes: CI = 95% confidence interval with lower and upper limits, **, *** = significant values, and S.E = standard error.

## Data Availability

Data may be provided on a reasonable request by contacting the corresponding authors.

## Appendix A. The Items Used in the Survey

Inclusive Leadership
Our manager is open to hearing new ideas.
Our manager is attentive to new opportunities to improve work processes.
Our manager is open to discuss the desired goals and new ways to achieve them.
Our manager is available for consultation on problems.
Our manager is an ongoing ‘presence’ in this team—someone who is readily available.
Our manager is available for professional questions I would like to consult with him/her.
Our manager is ready to listen to my requests.
Creativity
Employees here try to be as creative as they can.
Employees experiment with new approaches in performing their job.
When new trends develop, employees are usually the first to get on board.
Employees think creatively in performing their job.
Employees are inventive in overcoming barriers.
Polychronicity
I prefer to do two or more activities at the same time.
I typically do two or more activities at the same time.
Doing two or more activities at the same time is the most efficient way to use my time.
I am comfortable doing more than one activity at the same time.
I like to juggle two or more activities at the same time.
Psychological Safety
I am able to bring up problems and tough issues.
It is safe to take a risk in this hosptial.
It is easy for me to ask other members of this hosptial for help.
No one in this hospital would deliberately act in a way that undermines my efforts.
People in this hosptial sometimes reject others for being different.
